# Bilateral Acute Angle-Closure Glaucoma: A Case Report of an Unusual Cause of Acute Headache in a Child

**DOI:** 10.5811/cpcem.2021.7.52671

**Published:** 2021-09-09

**Authors:** Breelan Kear, Claudia R. Gold, Rahul Bhola

**Affiliations:** * Providence St. Joseph Hospital Orange, Department of Emergency Medicine, Orange, California; † CHOC Children’s Hospital, Department of Pediatric Emergency Medicine, Orange, California

**Keywords:** pediatrics, acute angle closure glaucoma, retinopathy of prematurity, case report

## Abstract

**Introduction:**

Acute angle-closure glaucoma (AACG) is typically considered a disease of adulthood. However, AACG may occasionally be seen in children. The clinical presentation is similar to adults, including headache, vomiting, and eye pain. However, the etiology of angle closure in children is different and most often associated with congenital anterior segment abnormalities. A precipitating factor of AACG in children with previous established, anterior segment abnormalities is eye dilation, which may occur during routine ophthalmological examination with topical mydriasis, or physiologic mydriasis upon entering a dark room.

**Case Report:**

We describe a 5-year-old child with a history of severe prematurity and retinopathy of prematurity (ROP) presenting with bilateral AACG following a routine outpatient, dilated ophthalmological examination. While angle-closure glaucoma has previously been reported in cases of ROP, a bilateral acute attack of AACG following pupil dilation in regressed ROP has hitherto been unreported.

**Conclusion:**

Given the association of ROP and AACG, it can be expected that as the survival rate of premature infants improves, the incidence of ROP and AACG may also increase. It is therefore prudent for the emergency physician to have AACG on the differential for pediatric patients with headache and eye pain.

## INTRODUCTION

Glaucoma is a leading cause of preventable blindness in the United States.^1^ The typical age demographic of glaucoma is older adults, peaking between ages 55–70.^2^ However, acute angle-closure glaucoma (AACG) can occur in any age group, particularly in those individuals with an anatomical predisposition that promotes angle closure.^3^ Retinopathy of prematurity (ROP) and eye dilation are known risk factors for developing AACG in childhood.^2^–^5^ Retinopathy of prematurity is most common in infants with a gestational age of fewer than 30 weeks.^2^ In pediatric patients, the clinical presentation of headache, vomiting, and eye pain may initially prompt one to consider non-accidental head trauma, migraine, infection, or intracranial mass in the differential. However, when evaluating these patients it is prudent for the emergency physician to be aware of the possibility of AACG, particularly in those patients with a history of severe prematurity. Given the advances in neonatology leading to higher survival rates in premature infants, there is now an increased incidence of ROP. This may potentially lead to more cases of pediatric AACG in the future.^5^

## CASE REPORT

This case describes headache, eye pain, and vomiting in a pediatric patient due to bilateral AACG. A 5-year-old boy with a history of extreme prematurity (23 weeks’ gestation), presented to the emergency department (ED) with his mother, reporting three days of bilateral eye pain with photophobia, headache, vomiting, and warmth to touch. She noted increased tearing but denied nasal congestion, runny nose, cough, earache, or sore throat. There was no known exposure to infectious disease, nor any known head or eye injury. There was no home temperature measured.

The patient’s past medical history was significant for global development delay, chronic lung disease, epilepsy, strabismus, and severe ROP. He had received laser treatment for retinopathy in infancy and successful strabismus surgery eight months prior to the ED visit. Four days previously, he had been evaluated in the ophthalmology clinic for his regular comprehensive ophthalmological exam. His eyes were dilated at that time as per prior routine (using cyclogyl, tropicamide, and phenylephrine spray) to facilitate dilated fundus exam. No other procedures were performed during that visit, and the ophthalmological exam was stable from prior examinations.

In the ED he was observed to be in significant distress. He was alert, agitated, and uncooperative with examination attempts. Vital signs were normal. There was no evidence of head or facial trauma. Topical proparacaine was used to help facilitate an eye exam; however, he tightly clenched his eyes and vigorously resisted all attempts to open them. Upon limited inspection, he had bilateral conjunctival injection without discharge or hemorrhage. His pupils were midsize and minimally reactive. His corneas appeared cloudy with no obvious abrasions or ulcerations. The remainder of the physical exam was unremarkable, and his mother confirmed he was at baseline neurological status.

The differential diagnosis following limited eye examination included acute iritis, keratitis, and AACG. There was minimal suspicion for infection, corneal abrasions, or ulcerations. We quickly recognized that a more thorough examination with procedural sedation would be required. An ophthalmology consultation was requested immediately. The patient was successfully sedated with intravenous (IV) ketamine. Under sedation, evaluation revealed bilateral corneal haziness, 2+ conjunctival injection, and both centrally and peripherally shallow anterior chambers. Intraocular pressures (IOP) were measured at 44 millimeters of mercury (mm Hg) (reference range: 12–22 mm Hg) oculus sinister (OS), and unrecordable oculus dexter (OD). The OD globe was significantly hardened on digital palpation. All findings pointed to the diagnosis of bilateral AACG.

Topical timolol/dorzolamide drops and IV acetazolamide were administered immediately following ophthalmology examination. Over the next two hours serial pressure measurements were taken with the tonometer and eyedrops were continued by the ophthalmologist in the ED. The patient was more sedated and therefore more cooperative with these serial exams. Intravenous mannitol was added. With this medical regimen, IOPs decreased from 44 mm Hg to 28 mm Hg OS and decreased from unmeasurable to 40 mm Hg OD. The patient was then admitted for observation and scheduled for surgical iridectomies in the morning.

Bromidine drops were ordered on admission. Upon re-evaluation the next morning, he remained in considerable discomfort, though pressures at that time were found to be much lower; 30 mm Hg OD, 14 mm Hg OS.

**CPC-EM Capsule**

What do we already know about this clinical entity?

*Acute angle closure glaucoma (AACG) is a common cause of blindness and eye pain in adults. However, it can also occur in children.*

What makes this presentation of disease reportable?

*The report describes a rare case of bilateral AACG in a child with a history of retinopathy of prematurity after a routine dilated fundoscopic exam.*

What is the major learning point?

*AACG can occur in children. Risk factors for AACG in children include retinopathy of prematurity, pupil dilation during fundoscopic examination, and certain systemic medications.*

How might this improve emergency medicine practice?

*It is important to keep AACG on the differential for children with headache, eye pain, and vision loss, especially in formerly premature infants where retinopathy of prematurity is common.*

Plans were initiated for more thorough examination under anesthesia, including gonioscopy and B-scan ultrasonography, followed by peripheral iridectomy of the right eye, and possibly the left. The ultrasound biomicroscopy of the right eye, obtained by the ophthalmologist under anesthesia, demonstrates the anterior iris insertion with anteriorly positioned ciliary body that predisposed the patient to AACG (Image). The patient was successfully treated with peripheral right iridectomy and discharged home post surgery without complications. He returned the following day for outpatient left peripheral iridectomy, which was also successful. His progress has been followed in ophthalmology clinic and he continues to do well. No further mydriatic drops have been used to facilitate examinations.

## DISCUSSION

Acute angle-closure glaucoma occurs when the angle of the anterior chamber of the eye is reduced and the trabecular meshwork of the iris is blocked, leading to an obstruction of the aqueous humor out of the anterior chamber.^3^,^6^ This leads to elevated IOP, causing severe pain and visual compromise, which can lead to blindness if left untreated.^6^ Shallow anterior chamber, farsightedness, and eyes with lens abnormalities are more susceptible to AACG.^6^ Retinopathy of prematurity is thought to be a risk factor, possibly due to the anatomical anterior displacement of the lens-iris diaphragm that effectively creates a shallow anterior chamber.^2^,^4^,^5^ An acute attack of glaucoma can be caused by pupillary dilation (such as entering into a dark room) or, as in the case of our patient, pharmacologic mydriasis during an eye exam.^5^–^7^

Although the clinical presentation of AACG in children is similar to adults, it is much more difficult to diagnose. This is primarily due to the limited ability of a young child to communicate history and cooperate with a thorough examination. In pediatric patients it may be necessary to both examine and treat the eye under procedural sedation, as occurred in our case.^5^ We used ketamine for sedation as it was the most routinely used in our pediatric ED. It is worth noting that ketamine has been thought to increase IOP; however, the evidence for this is poor.^8^–^10^ More recent small studies have measured IOPs of patients with non-ophthalmologic injuries undergoing procedural sedation with ketamine. These studies suggest that there is no increase in IOP or that the increase is negligible.^8^–^10^ However, until larger studies are performed it would have been reasonable to use another medication during sedation that is not theoretically associated with increased IOP.

The classic findings of AACG are unilateral eye redness with a non-reactive, mid-dilated pupil, as well as corneal haziness and high IOP. Patients usually have significant eye pain, vision changes, headache, nausea, and vomiting.^6^ The vast majority of AACG affects only one eye. It is exceedingly rare to have bilateral AACG.^11^ Only one other case report of bilateral AACG from pharmacologic mydriasis has been described in a three-year-old child, who similarly presented with AACG four days following a dilated eye examination.^7^ Pediatric bilateral AACG has also been reported as a result of systemic medications such as lisdexamfetamine dimesylate,^12^ carbamazepine,^13^ and topiramte,^14^ although this is still exceedingly rare. 12–14

The immediate management of AACG includes decreasing aqueous humor production. This is achieved through the use of ophthalmic timolol drops, a non-selective beta blocker, as well as IV acetazolamide, a carbonic anhydrase inhibitor. In severe cases, IV mannitol also may be used to create an osmotic gradient between the blood and the vitreous to lower vitreous volume.^6^,^15^ An alpha-adrenergic agonist, such as apraclonidine or bromidine drops, is used to decrease aqueous production.^15^ Miotic drops, such as pilocarpine, are sometimes used to reopen the angle. These are thought to be ineffective at very high pressures (above 40–50 mm HG) and are sometimes reserved for use once the pressure improves.^6^,^15^ Ultimately, the treatment is iridotomy by an ophthalmologist.

## CONCLUSION

Although acute angle-closure glaucoma is typically considered a disease of older adults, we describe a case of bilateral AACG in a 5-year-old male. This case highlights the importance of keeping AACG on the differential in pediatric patients who present with headache, eye pain, and vomiting. Risk factors include a history of retinopathy of prematurity or recent pharmacological eye dilation. The physical exam findings are paramount in the diagnosis. Sedation may be required to adequately examine the eye, which could inhibit prompt diagnosis. However, early recognition and treatment of AACG in pediatric patients will prevent vision loss.

## Figures and Tables

**Image:**
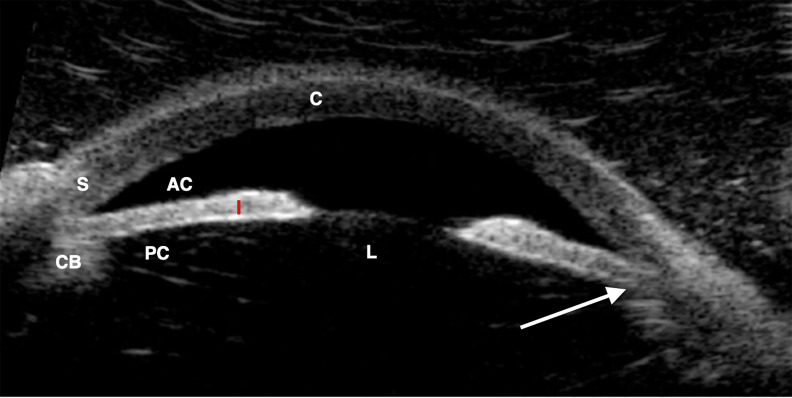
Ultrasound biomicroscopy of right eye showing anterior iris insertion with anteriorly positioned ciliary body (arrow). The anterior chamber (AC), ciliary body (CB), cornea (C), iris (I), lens (L), posterior chamber (PC), and sclera (S) are annotated.
